# Changes in the Number of Double-Strand DNA Breaks in Chinese Hamster V79 Cells Exposed to γ-Radiation with Different Dose Rates

**DOI:** 10.3390/ijms140713719

**Published:** 2013-07-01

**Authors:** Konstantin V. Kotenko, Andrey Y. Bushmanov, Ivan V. Ozerov, Denis V. Guryev, Natalya A. Anchishkina, Nadezhda M. Smetanina, Ekaterina Y. Arkhangelskaya, Natalya Y. Vorobyeva, Andreyan N. Osipov

**Affiliations:** State Research Center—Burnasyan Federal Medical Biophysical Center of Federal Medical Biological Agency (SRC-FMBC), 46, Zhivopisnaya Str, Moscow 123098, Russia; E-Mails: fmbc-fmba@bk.ru (K.V.K.); radclin@yandex.ru (A.Y.B.); varnivey@gmail.com (I.V.O.); denis.guryev@gmail.com (D.V.G.); nanchishkina@gmail.com (N.A.A.); smetaninanm@gmail.com (N.M.S.); ek.arkhangelskaya@gmail.com (E.Y.A.); nuv.rad@mail.ru (N.Y.V.)

**Keywords:** DNA double-strand breaks, γ-H2AX foci, γ-radiation, dose rate effect, V79 cells

## Abstract

A comparative investigation of the induction of double-strand DNA breaks (DSBs) in the Chinese hamster V79 cells by γ-radiation at dose rates of 1, 10 and 400 mGy/min (doses ranged from 0.36 to 4.32 Gy) was performed. The acute radiation exposure at a dose rate of 400 mGy/min resulted in the linear dose-dependent increase of the γ-H2AX foci formation. The dose-response curve for the acute exposure was well described by a linear function *y* = 1.22 + 19.7*x*, where “*y*” is an average number of γ-H2AX foci per a cell and “*x*” is the absorbed dose (Gy). The dose rate reduction down to 10 mGy/min lead to a decreased number of γ-H2AX foci, as well as to a change of the dose-response relationship. Thus, the foci number up to 1.44 Gy increased and reached the “plateau” area between 1.44 and 4.32 Gy. There was only a slight increase of the γ-H2AX foci number (up to 7) in cells after the protracted exposure (up to 72 h) to ionizing radiation at a dose rate of 1 mGy/min. Similar effects of the varying dose rates were obtained when DNA damage was assessed using the comet assay. In general, our results show that the reduction of the radiation dose rate resulted in a significant decrease of DSBs per cell per an absorbed dose.

## 1. Introduction

The majority of cellular DNA lesions caused by ionizing radiation (IR) significantly differ from those caused by endogenous sources in their physical and chemical properties [1]. The most important features of radiation-induced DNA lesions are their complexity and clustering [2]. The radiation-induced DNA double-strand breaks (DSBs) are the most crucial for the cell fate. Thus, DSBs are assumed to be a basic trigger for the main processes of cellular response to the radiation exposure [3]. Being quite a slow process, repair of DSBs may be inefficient and may result in serious cytogenetic lesions, cell death, the tumor suppressor genes inactivation or an activation of certain oncogenes [4,5]. The principal mechanisms of DSBs induction and repair after an acute and short-term exposure to IR at different doses have been well studied [6–9]. However, in environmental conditions, living organisms are continuously exposed to low dose rate IR. Unfortunately, the reports describe the induction and repair of DSBs after chronic exposure to IR in mammalian cells are contradictory. Thereby, there is still no clear understanding as to whether the mechanisms of the induction and repair of DSBs under chronic IR exposures are similar to those described for acute exposures in the same dose region.

The goal of this study was to measure and compare the formation of DSBs in Chinese hamster V79 cells after the exposure to γ-radiation at various dose rates. The immunocytochemical analysis of the phosphorylated H2AX-histone (γ-H2AX) foci was performed to estimate the cellular DNA DSBs formation. ATM, ATR and DNA-PK, are the major kinases that phosphorylate H2AX following DSB [10]. As a result, dynamic microstructures (foci) with thousands of γ-H2AX molecules are formed at each DSB [11]. One γ-H2AX focus is considered to be associated with one DSB [12]. In addition, we used the neutral version of the single cell gel-electrophoresis method (comet assay) to estimate the double-stranded DNA fragmentation [13].

## 2. Results and Discussion

**Figure 1:**
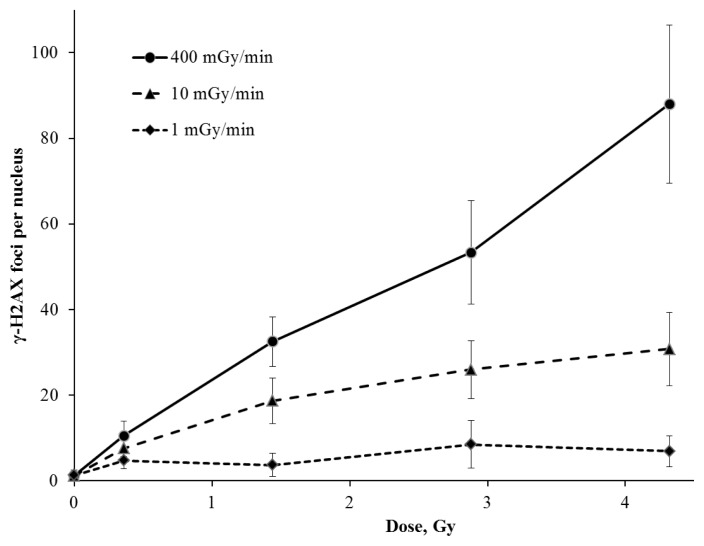
The yield of γ-H2AX foci in Chinese hamster V79 cells exposed to γ-rays at high (400 mGy/min), medium (10 mGy/min) and low (1 mGy/min) dose rates.

Figure 1 shows the formation of γ-H2AX foci per cell nucleus after acute (400 mGy/min) and protracted (1 and 10 mGy/min) irradiation with doses ranging between 0.12 and 4.32 Gy. The γ-H2AX foci measurement was carried out 30 min after the acute exposure to IR. The resulting dose response curve for γ-H2AX formation was linear (Figure 1) and was well described by the function *y* = 1.2 + 19.7*x* (*R*^2^ = 0.99) where “*y*” is an average number of γ-H2AX foci per cell nucleus and “*x*” is an absorbed dose (Gy). Assuming that a single focus is associated with a single DNA double-strand break, our results indicate that the acute exposure of V79 cells to IR induced approximately 20 DNA DSBs per cell per Gy. The results obtained are consistent with the literature data on DSB induction in mammalian cells by sparsely IR [14]. However, it is worth mentioning that the actual number of DSBs per cell produced by 1 Gy of IR may be higher than the observed number of foci; two or more closely located DSBs may be processed by DNA repair machinery and be joined into a single larger focus that can be taken into account as a single DSB.

Here we demonstrate that the exposure to IR at a lower dose rate (10 mGy/min) leads to a reduced number of γ-H2AX foci per cell nucleus. The foci number increases at the dose of 1.44 Gy and reaches “plateau” at doses ranged from 1.44 to 4.32 Gy (Figure 1). The dose-response curve obtained shows the result of two simultaneously occurring processes in cells exposed at a low dose rate: DSBs induction and their repair. In fact, mammalian cells use two major pathways to repair DNA DSBs, which are non-homologous end joining (NHEJ) and homologous recombination (HR) [15]. Hereby, the most of DSBs (approximately 75%) are repaired by the relatively fast NHEJ pathway [16]. The number of radiation-induced DSBs was found to decrease two-fold during 30 min following the exposure [17]. In case of the prolonged irradiation at the same dose rate, there was a certain balance between DSBs formation and their repair (approximately 12 DSBs per cell per h) and the “plateau” effect appeared.

**Figure 2:**
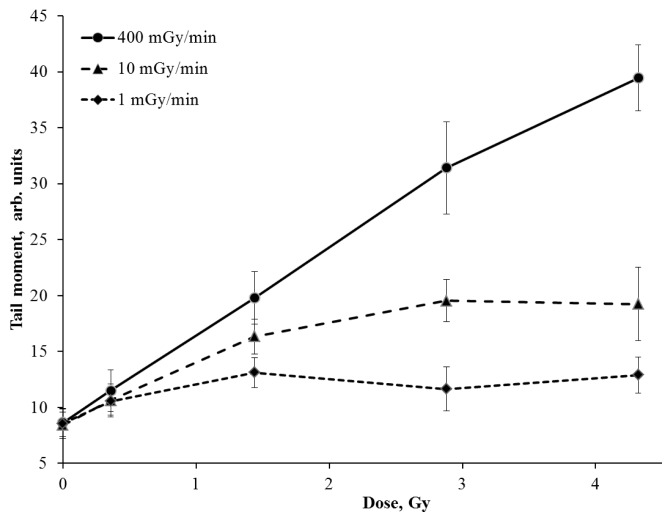
Changes in the DNA fragmentation level in Chinese hamster V79 cells exposed to γ-rays at high (400 mGy/min), medium (10 mGy/min) and low (1 mGy/min) dose rates.

Interesting results on cells exposed to IR at the dose rate of 1 mGy/min have been obtained. There was only a slight increase of the γ-H2AX foci number (up to 7) during all irradiation time (up to 72 h) (Figure 1). Recently, Japanese researchers reported similar results obtained on human fibroblasts exposed to IR at doses up to 5 Gy with a dose rate of 0.3 mGy/min. There was no increase in the level of the phosphorylated p53 protein and the γ-H2AX foci number was only slightly elevated in comparison with control values [18]. However, there is no consensus on the mechanisms of this phenomenon in the literature. It has been reported that the ATM-kinase activation in cultured mammalian cells exposed to IR at a dose rate of 1.5 mGy/min (up to 6 Gy) is reduced [19]. The authors explained the effect of the significantly lower number of γ-H2AX foci (corresponding to the radiation dose) in cells exposed to IR at a low dose rate by the failure to activate ATM-associated repair pathways. However, there is some evidence that the phosphorylation of H2AX-histone in cells irradiated at a low dose rate is an ATM-independent process [20]. Moreover, the accumulation of unrepaired breaks should increase the chromatin fragmentation level. To confirm this hypothesis we have estimated the DNA fragmentation level induced by DSBs using the neutral version of comet assay. These results are shown in Figure 2. The changes in the DNA fragmentation level in V79 cells irradiated with different dose rates are found to correlate with the yield of γ-H2AX foci. Moreover, the reduced dose rate of IR resulted in a significant decrease of the DNA fragmentation level (Figure 2).

Thereby, we propose that an effective DSBs repair and H2AX-histone dephosphorylation take place in normal mammalian cells (without DNA repair deficiency) exposed to low dose and low dose rate sparsely IR. This speculation is based on the results of the comparative study on the cells exposed to IR with different dose rates. As shown earlier in TERT-immortalized human fibroblast, the cytogenetic lesions frequency as well as an elimination of cells irradiated at a low dose rate (0.3 mGy/min) were significantly lower than those in cells irradiated at a high dose rate (2000 mGy/min) [21]. Similar effects were observed in the animal model experiments [22–24].

According to the calculations made by Vilenchik and Knudson, the yield of endogenously-induced DSBs in proliferating human fibroblasts is the same as for the cells irradiated at a dose rate of 5 mGy/min (~8 DSBs/cell/h) [25]. Thus, radiation-induced DSBs can be repaired easily in cells exposed to IR at a dose rate of 1 mGy/min. Our data confirms this conclusion, however there are still some questions to be answered: (1) are the processes of both the endogenous and low-dose radiation-induced DSBs repair are similar and what are the distinctions between them? (2) Might the DNA microdeletions formed by the NHEJ repair pathway lead to the genome instability and the oncological transformation of cells? Our further studies are aimed to find the answers.

## 3. Experimental Section

### 3.1. Cell Line and Culture Technique

Chinese hamster V79 lung fibroblasts obtained from the American Type Culture Collection were maintained with twice weekly subculture in DMEM supplemented with 10% foetal bovine serum (FBS), 100 mg/mL penicillin/streptomycin and 1% l-glutamine. They were grown at 37 °C in the presence of 5% CO_2_.

### 3.2. Cell Irradiation

Exponentially growing cells in concentration of 2 × 10^5^ cells per milliliter of medium were plated down into T-25 flasks and maintained at 37 °C in the presence of 5% CO_2_ for 24 h prior to irradiation. Irradiation with Co^60^ rays was performed in the specially designed facility for cell culture irradiation (guided temperature conditions with the presence of 5% CO_2_) with measured dose rates of 0.1, 1, 10 and 400 mGy/min. The total absorbed doses were 0.36, 1.44, 2.88 and 4.32 Gy. The IR exposure times of 0.9, 3.6, 7.2, 10.8 min at the dose rate of 400 mGy/min, 0.6, 2.4, 4.8, 7.2 h at the dose rate of 10 mGy/min and 6, 24, 48 and 72 h at the dose rate of 1 mGy/min were chosen respect ively.

### 3.3. Immunocytochemistry for γ-H2AX

To analyze γ-H2AX foci, the method previously described was used [26]. Shortly, cells grown on coverslips were fixed for 20 min in 2% freshly prepared paraformaldehyde, rinsed by tris-buffer and permeabilized in cool (−20 °C) methanol for 1 min. Then cells were transferred into tris-buffer containing 4% FBS and 0.1% Triton X-100 for 20 min. Slides were incubated with monoclonal antibodies against γ-H2AX (Anti-phospho-Histone H2AX Rabbit Monoclonal, Merck-Millipore, Darmstadt, Germany) at 4 °C over night. Then cells were rinsed and incubated with fluorochrome-conjugated secondary antibodies (Goat anti-Rabbit IgG (H + L), FITC conjugate, Merck-Millipore) at room temperature for 1 h. DNA was stained with DAPI (0.5 μg/mL, 5 min). The visualization and further analysis of images were performed using fluorescent microscope Axioscop-40 FL (Carl Zeiss, Oberkochen, Germany) equipped with the high-resolution camera AxioCamMRc 5 (Carl Zeiss, Oberkochen, Germany) and AxioVision 4.8 software (Carl Zeiss, Oberkochen, Germany). The foci were typically scored manually in a blinded manner. One hundred cells per slide were analyzed.

### 3.4. DNA Comet Assay

The level of DNA fragmentation as a result of DSBs formation was estimated by a modified neutral version of the comet assay. [13]. According to the method, the number of DSBs was proportional to the extent of DNA migrated away from the nucleus during electrophoresis of agarose-immobilized single cells. Briefly, 10 μL of cell suspension (1 × 10^5^ cells/mL) was mixed with 100 μL of 0.5% low melting agarose (Sigma Chemical, St. Louis, MO, USA) in PBS (pH 7.4) at 37 °C and 75 μL was layered on top of the microscope slides pre-coated with 1% normal agarose (Sigma Chemical, St. Louis, MO, USA). The slides were covered by coverslips and incubated for 5 min at 4 °C. After removing coverslips and following a 2 h lysis (2.5 M NaCl, 100 mM EDTA, 10 mM Tris-HCl, pH 10.0, 1% Triton X-100, 10% DMSO) at 4 °C, the slides were incubated 20 min in TBE buffer (89 mM Tris base pH 8.0, 89 mM boric acid, 2 mM EDTA) at 4 °C. The electrophoresis was carried out in TBE buffer at 1.5 V/cm and 4 °C for 20 min. The slides were then fixed (70% ethanol for 5 min) and dried.

Fluorescent dye SYBRGreen I was utilized to visualize DNA. Analysis of comets was performed using the Axioscop-40 FL microscope (Carl Zeiss, Oberkochen, Germany) equipped with the high-resolution digital camera AxioCamMRc 5 (Carl Zeiss) and AxioVision 4.8 software (Carl Zeiss). Images were analyzed using the specially designed CASP 1.2.2 software (CASPlab, Wroclaw, Poland).

### 3.5. Statistics

Statistical errors were calculated as the standard error. All the experiments were performed at least three times and the data was presented as the mean value ± standard error in each case.

## 4. Conclusions

The present study of the radiation-induced DSBs level in Chinese hamster V79 cells exposed to IR at various dose rates showed a significant decrease in the DSBs level per IR dose in cells irradiated at a lower dose rate compared to a higher dose rate. Evidently, current findings support the threshold hypothesis on the biological effects of IR. The dose rate appeared to be an essential factor in cellular response to IR determining biological effects induction and realization, probably more important than the absorbed dose. It is therefore advisable to consider the dose rate factor in the radiological protection standards.
